# Sex differences in long-term kidney fibrosis following neonatal nephron loss during ongoing nephrogenesis

**DOI:** 10.1186/s40348-023-00164-4

**Published:** 2023-08-25

**Authors:** Carlos Menendez-Castro, Nada Cordasic, Fabian B. Fahlbusch, Joachim Woelfle, Karl F. Hilgers, Andrea Hartner

**Affiliations:** 1 Department of Pediatrics and Adolescent Medicine, University Hospital of Erlangen, Erlangen, Germany; 2 Department of Nephrology and Hypertension, University Hospital of Erlangen, Erlangen, Germany; 3 Division of Neonatology and Pediatric Intensive Care Medicine, University Hospital of Erlangen, Erlangen, Germany

**Keywords:** Sex differences, Kidney fibrosis, Neonatal nephron loss, Nephrogenesis, Neonatal uninephrectomy

## Abstract

**Background:**

Clinical studies suggest that female sex plays a protective role in the development and progression of kidney disease. Recent experimental studies indicate that in male rats early nephron loss under ongoing nephrogenesis is accompanied by severe long-term sequelae. In humans, nephron formation occurs mainly in the third trimester, ceasing with 36 weeks of gestation. Due to perinatal complications, preterm infants delivered during this vulnerable period may undergo acute nephron loss. In rats nephrogenesis persists until postnatal day 10, reflecting the situation of human preterms with persisting nephrogenesis. In our animal model of neonatal uninephrectomy, female and male rats were uninephrectomized at day 1 of life. Hypothesizing sex-dependent differences, long-term renal outcome was assessed after 1 year.

**Results:**

In both sexes, neonatal uninephrectomy was not followed by arterial hypertension at 1 year of age. Compensatory weight gain and glomerular hypertrophy of the remaining kidney occurred in uninephrectomized female and male animals. Selected markers of interstitial inflammation and fibrosis were regulated sex-dependently. The expression of monocyte chemoattractant protein-1 was increased in females, while tubulointerstitial infiltration by M1 macrophages was significantly higher in males after neonatal uninephrectomy. Neonatally uninephrectomized male rats had more glomerulosclerosis and podocyte damage compared to females, which was assessed by a semiquantitative score and desmin staining. RT-PCR revealed that after neonatal uninephrectomy in the remaining contralateral kidney of female rats the expression of candidate genes of renal development and function, i.e., *wt-1, nephrin, synaptopodin*, *gdnf*, and *itga8* was higher than in males.

**Conclusions:**

Based on these observations we conclude that female sex is protective in the long-term response of the kidney to acute nephron loss under active nephrogenesis.

**Supplementary Information:**

The online version contains supplementary material available at 10.1186/s40348-023-00164-4.

## Background

The development and progression of renal disease is sex-dependent [1, 2]. Several clinical studies highlight the fact that female sex is protective for renal function, e.g., female sex slows eGFR decline or progression to end stage renal disease [3] or development of renal fibrosis [4]. This notion is supported by animal studies. Uninephrectomy in adult rats led to tubular and glomerular damage in male, not in female rats after 2 months [5]. Moreover, a reduction of renal mass in a rat model of uninephrectomy at the age of 6 weeks and postinterventional high salt intake over 2 weeks male animals showed more severe kidney injury at the age of 18 months compared to females [6]. The consequences of a nephron loss during ongoing nephrogenesis and before the onset of puberty for male and female individuals, however, are not yet clear. In preterm neonates prior to 36 weeks of gestation, nephrogenesis is still active. Immature kidneys are highly vulnerable with an increased risk to early nephron loss [7] due to hypoxic-ischemic injury or adverse drug effects [8].

Recent clinical studies underline the link between acute neonatal nephron loss and secondary renal and cardiovascular disease later in life [9–11]. Animal studies suggest glomerular hypertrophy to be a central pathogenic factor of progressive kidney injury after acute nephron loss [12, 13]. Furthermore, nephron loss is often followed by compensatory glomerular hyperfiltration and systemic hypertension, which in turn leads to glomerular damage and promotes cardiovascular disease [14, 15].

With our animal model of neonatal uninephrectomy at day 1 of life, we took advantage of the fact that rats show a still active nephrogenesis until day 10 after birth [16]. Thus, our model resembles to some degree the situation of preterm neonates suffering from acute nephron loss during ongoing organogenesis. Our previous studies in this animal model showed that early nephron loss is followed by significantly altered expression levels of central molecular markers of kidney homeostasis and integrity in male animals [17]. Moreover, uninephrectomy at day 1 of life led to structural and functional changes found in the remaining kidneys of 1-year-old male rats [18]. In this study, we addressed the question whether female sex is still protective in our rat model of early neonatal nephron loss.

## Materials and methods

### Animal procedures

All animal experimentation was performed in compliance with the Directive 2010/63/EU of the European Parliament and was approved by the local government authorities (Regierung von Mittelfranken, AZ No. 54.2532.1–24/10 and Regierung von Unterfranken, AZ No. 55.2.2–2532-2–526). Six pregnant female Wistar rats received standard rodent chow (ssniff Spezialdiäten GmbH, Soest, Germany) with free access to tap water in a room maintained at 22 ± 2 °C with a 12-h dark/light cycle. After spontaneous delivery, male and female pups from 6 different litters were either uninephrectomized at day 1 of life as described before, or were sham operated as a control [18]. At the age of 1 year, 8 male uninephrectomized and 8 male control rats, as well as 6 female uninephrectomized and 10 female control rats were sacrificed by bleeding in anesthesia.

### Blood pressure measurements

Intraarterial blood pressure measurements were obtained at the day of sacrifice, as described in detail [19, 20]. In short, catheters were implanted in the right femoral artery of anesthesized rats. After a recovery phase of 2 h, blood pressure was recorded by a polygraph (Hellige, Freiburg, Germany) in conscious rats for 30 min.

### Serum and urine analyses

One day before sacrifice, rats were put in metabolic cages for 24 h for urine collection. Proteinuria was assessed using Bio-Rad Protein Assay (Bio-Rad, Feldkirchen, Germany). Blood samples were obtained before sacrifice under isoflurane anesthesia. Plasma creatinine, urea and phosphate were measured using the automatic analyser Integra 1000 (Roche Diagnostic, Mannheim, Germany).

### Tissue preparation

Immediately after sacrifice, rats were weighted, kidneys were excised, decapsulated and weighted. Both poles of the kidneys were snap frozen in liquid nitrogen for RNA analysis, the other part was cut transversally in the center of the kidney, fixed in methyl-Carnoy’s solution and embedded in paraffin for histological analyses or immunohistochemistry.

### Histological analyses

Two micrometer sections were cut transversally from the central cross section and stained with periodic acid-Schiff’s reagent (PAS) and counterstained with hematoxylin. In PAS-stained sections, glomerular perimeters were measured in 50 glomeruli per section using Metavue software (Metavue, Molecular Devices, Sunnyvale, CA, USA). Glomeruli included in the analysis were evenly distributed over the section, sparing very marginally cut glomeruli (< 30 µm diameter). A semiquantitative score for glomerulosclerosis was used as described before [18].

### Immunohistochemistry

M1 Macrophages/monocytes were counted after staining for the rat macrophage/monocyte marker ED-1 as described previously [18]. Interstitial ED-1-positive cells were counted in 20 cortical views (magnification × 250) per section and expressed as cells per medium-power field. M2 macrophages were stained using anti-CD 163. CD 163-positive cells were counted in 40 medium-power cortical views per section after staining with anti-CD163 (Abcam, Cambridge, UK). Desmin staining (monoclonal antibody by DAKO, Hamburg, Germany) was evaluated as a parameter of podocyte injury. For evaluation of desmin immunoreactivity, a score of 0 to 4, based on the stained area of the glomerulus, was used. At least 30 glomeruli per section were evaluated. The degree of interstitial fibrosis was determined by evaluation of collagen I staining [21]. Immunohistochemistry for collagen I was performed with a rabbit polyclonal antibody to collagen I (Biogenesis, Poole, England), as described previously. Interstitial collagen I was quantified in 30 medium-power views using an 11 × 11 point grid. The percentage of grid points corresponding with a stained area was calculated [21]. All histological evaluations were done in renal tissue from 10 control females, 6 UNX females, 8 control males and 8 UNX males and were performed by a single investigator blinded to the group assignment.

### Real-time PCR analyses

PCR analyses were done in renal tissue from 10 control females, 6 UNX females, 8 control males, and 8 UNX males. Frozen kidney tissue was homogenized in RLT buffer reagent (Qiagen, Hilden, Germany) with an Ultra-Turrax for 30 s and total RNA was extracted with RNeasy® Mini columns (Qiagen) according to the manufacturer’s instructions. TaqMan reverse transcription reagents (Applied Biosystems, Waltham, MA, USA) with random hexamers as primers were used to obtain first-strand cDNA. Final RNA concentration in the reaction mixture was adjusted to 0.1 ng/µl. To test for genomic DNA contamination, reactions without Multiscribe reverse transcriptase were performed as negative controls. Reverse transcription products were diluted 1∶1 with dH_2_O. Then, real-time PCR was performed with an ABI PRISM 7000 Sequence Detector System and SYBR Green (Applied Biosystems) or TaqMan reagents (Applied Biosystems) according to the manufacturer’s protocol. The relative amount of the specific mRNA was normalized with respect to 18S rRNA. See supplementary data (Supplemental Table S1) for primers and probes used for amplification. Primer pairs were designed using the Primer Express software (Perkin Elmer, Foster City, CA, USA). mRNA levels were calculated and normalized to a housekeeping gene (18S) with the ∆-∆-C_T_ method as specified by the manufacturer (https://assets.thermofisher.com/TFS-Assets/LSG/manuals/cms_040980.pdf).

### Analysis of data

Data are expressed as mean ± standard error of the mean (SEM). After testing for normality distribution, one-way analysis of variance (ANOVA) was performed, followed by Fisher’s least significant differences (LSD) post hoc test to assess the differences between the groups using the SPSS Statistics 19 software (IBM, Ehningen, Germany). Results were considered significant at *p* < 0.05.

## Results

Male and female neonatal rats were uninephrectomized at day 1 of life, resulting in a reduction of renal mass during ongoing nephrogenesis and before the onset of puberty. Renal structural and functional parameters of female and male rats were assessed 1 year after neonatal uninephrectomy.

### Auxologic data and specifications of renal structure and function

Body weight showed significant sex differences between both UNX animals and controls (Table 1). Uninephrectomy did not specifically affect body weight. Comparable values of mean arterial blood pressure were found among all experimental groups.

**Table 1:** Auxologic data and parameters of renal structure and function

	Male Co	Male UNX	Female Co	Female UNX
Body weight [g]	761.21 ± 22.57	743.01 ± 27.61	404.58 ± 13.84 ^§^	372.46 ± 30.4 ^§^
Right kidney weight [g]	1.90 ± 0.07	3.49 ± 0.22 *	1.25 ± 0.05 ^§^	2.05 ± 0.08 *^§^
Glomerular perimeter [µm]	362.1 ± 7.5	413.0 ± 5.7 *	310.9 ± 7.4 ^§^	364.1 ± 7.5 *^§^
Mean arterial pressure [mmHg]	118.8 ± 2.1	122.1 ± 4.2	110.73 ± 1.12	108.82 ± 2.65 ^§^
Serum creatinine [mg/dl]	0.31 ± 0.02	0.36 ± 0.02	0.26 ± 0.01 ^§^	0.25 ± 0.02 ^§^
Serum urea [mg/dl]	33.98 ± 1.57	38.73 ± 2.41	31.87 ± 1.26	29.76 ± 2.87 ^§^
Serum phosphate [mmol/l]	1.93 ± 0.12	1.99 ± 0.16	1.26 ± 0.07 ^§^	1.17 ± 0.06 ^§^
Proteinuria [mg/d]	37.78 ± 14.37	209.11 ± 52.47 *	92.21 ± 38.67	387.38 ± 132.09 *
*Kim-1* expression [fold change]	1.00 ± 0.32	4.48 ± 0.95	0.76 ± 0.31	7.76 ± 3.68 *
*Ngal* expression [fold change]	1.00 ± 0.11	4.20 ± 1.13	1.97 ± 0.38	10.42 ± 4.44 *^§^

*UNX* rat uninephrectomized at day 1 of life, *Co* age-matched sham-operated control

^*^p < 0.05 versus respective control

^§^*p* < 0.05 versus male

Kidney weights as well as glomerular perimeters were lower in female groups compared to respective male groups. Both female and male rats showed a significant compensatory gain of right kidney weight after uninephrectomy (Table 1). Glomerular perimeters in the remaining kidney were higher in uninephrectomized rats of both sexes compared to controls (Table 1). Serum creatinine level was significantly lower in females of control and UNX groups when compared to the respective male group. Values of serum urea were comparable in female and male controls and were significantly lower in UNX females compared to UNX males. Uninephrectomy led to a significant proteinuria in 52 weeks old females and males likewise (Table 1). On the other hand, expression levels of kidney injury molecule-1 *(kim-1)* and neutrophil gelatinase-associated lipocalin *(ngal)* as molecular markers of renal damage were significantly higher in UNX female rat only compared to controls, with a respective tendency in males (Table 1).

### Markers of kidney inflammation and fibrosis

Assessment of the chemokine monocyte chemoattractant protein-1 *(mcp-1)* showed an increased expression after uninephrectomy only in females, not in males (Table 2). The expression of the inflammatory marker osteopontin *(opn)* was induced in UNX males and females likewise (Table 2). Renal infiltration by M1 macrophages was significantly higher after uninephrectomy in males compared to controls (Table 2), while the infiltration of M2 macrophages remained unchanged following uninephrectomy in both sexes (Table 2).

**Table 2:** Markers of renal inflammation and fibrosis

	Male Co	Male UNX	Female Co	Female UNX
*Mcp-1* expression [fold change]	1.00 ± 0.14	1.61 ± 0.55	0.91 ± 0.15	2.82 ± 1.04 *
*Opn* expression [fold change]	1.00 ± 0.20	8.23 ± 2.82 *	1.59 ± 0.35	8.57 ± 3.26 *
M1 macrophages [No./MPF]	2.51 ± 0.53	6.25 ± 1.47 *	2.73 ± 0.24	5.05 ± 1.17
M2 macrophages [No./40 MPFs]	16.63 ± 5.42	13.00 ± 1.36	27–55 ± 6.43	35.25 ± 14.69
Collagen I stain [% positive area]	0.77 ± 0.11	1.94 ± 0.26 *	0.76 ± 0.06	0.87 ± 0.14 ^§^
*Collagen I* expression [fold change]	1.00 ± 0.10	2.70 ± 0.76 *	1.68 ± 0.16	3.05 ± 0.54 *
*Collagen IV* expression [fold change]	1.00 ± 0.06	1.69 ± 0.53	3.06 ± 0,50 ^§^	2.90 ± 0.59

*UNX* rat uninephrectomized at day 1 of life, *Co* age-matched sham-operated control, *MPF* medium power field

^*^p < 0.05 versus respective control

^§^*p* < 0.05 versus male

**Fig. 1:**
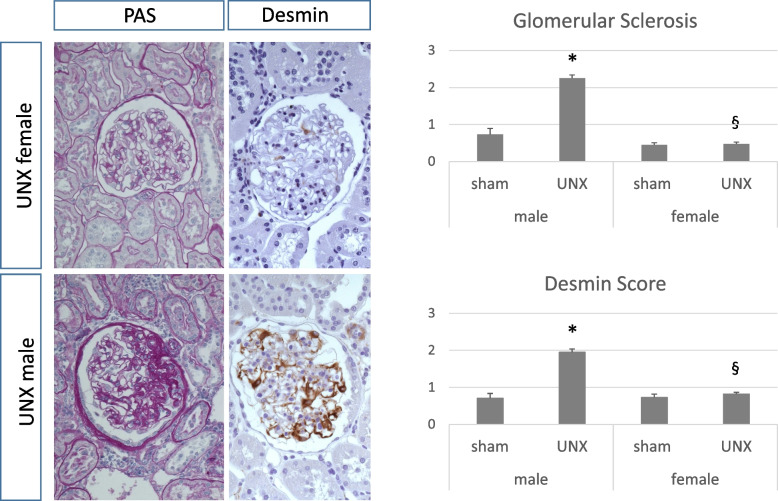
Glomerulosclerosis and podocyte damage 52 weeks after neonatal uninephrectomy. **A** Glomerulosclerosis score with representative photomicrographs assessed in PAS-stained glomeruli. **B** Glomerular desmin score with representative photomicrographs. UNX, rat uninephrectomized at day 1 of life. Sham, age-matched sham-operated control. Co male UNX *n* = 8, UNX male *n* = 8, Co female *n* = 10, UNX female *n* = 6. Data are mean ± SEM. * UNX male vs Co male *p* < 0.05.^§^ UNX male vs UNX female < 0.05 (one-way ANOVA, LSD *post-hoc* test)

Glomerulosclerosis, assessed by a semiquantitative score, was increased in UNX males only (shown in Fig. 1 and Supplemental Figure S1). Similarly, desmin-positive podocytes as markers of podocyte damage, became evident after uninephrectomy in males, not in females (Fig. 1). Interstitial deposition of collagen I was increased only in UNX males (Table 2, Supplemental Figure S2), while collagen I expression was induced in both female and male UNX rats (Table 2). Collagen IV expression levels were only increased in the female control group when compared to the male control group (Table 2).

### Markers of renal development and function

Expression of Wilms’ tumor suppressor gene *(wt-1)* and *nephrin*, as markers of podocyte integrity, was significantly reduced after uninephrectomy in both sexes. However, the expression of *wt-1* and *nephrin* was higher in the female control group as well as in female UNX compared to the respective male UNX (Table 3). *Synaptopodin*, glial cell line-derived neutrophic factor *(Gdnf)* and integrin alpha-8 *(Itga8)*, which are known to have renoprotective functions and to be involved in renal development, showed higher expression levels in females compared to males (Table 3).

**Table 3:** Markers of renal development and integrity

	Male Co	Male UNX	Female Co	Female UNX
*Wt-1* expression [fold change]	1.00 ± 0.07	0.70 ± 0.06 *	1.68 ± 0.10 ^§^	1.08 ± 0.10 *^§^
*Nephrin* expression [fold change]	1.00 ± 0.07	0.71 ± 0.07 *	1.60 ± 0.04 ^§^	1.25 ± 0.116 *^§^
*Synaptopodin* expression [fold change]	1.00 ± 0.08	1.04 ± 0.16	1.69 ± 0.08 ^§^	1.49 ± 0.12 ^§^
*Gdnf epxression* [fold change]	1.00 ± 0.11	0.68 ± 0.09	1.94 ± 0.16 ^§^	1.21 ± 0.16 *^§^
*Itga8* expression [fold change]	1.00 ± 0.04	1.00 ± 0.09	1.93 ± 0.09 ^§^	1.84 ± 0.29 ^§^

*UNX* rat uninephrectomized at day 1 of life, *Co* age-matched sham-operated control

^*^p < 0.05 versus respective control

^§^*p* < 0.05 versus male

## Discussion

In our study, we assessed kidney fibrosis 1 year after acute neonatal nephron loss during ongoing nephrogenesis in rats of both sexes. Our long-term data suggests that male animals suffer from more severe renal sequelae, similar to previous studies in human patients [22]. However, neonatal uninephrectomy did not result in subsequent arterial hypertension neither in female nor in male animals. Therefore, renal alterations have to be regarded as independent and not secondarily induced. As reported previously, the absence of arterial hypertension contrasts with the observations of Woods et al., showing arterial hypertension in female Sprague–Dawley rats after neonatal uninephrectomy and might be explained by species differences of experimental animals or the different approaches to assess blood pressure [18, 23].

Glomerular hypertrophy is one of the main alterations occurring within compensatory gain of renal mass following acute nephron loss [24]. In our study, both sexes showed increased glomerular diameters after uninephrectomy. Noteworthy, the glomerular size of UNX males was significantly higher than in UNX females. These observations are in line with Elsherbiny et al., who assessed renal alterations of living kidney donors and found a positive correlation between male sex and secondary glomerular hypertrophy [25]. In female and male rats compensatory renal growth and gain of glomerular volume after uninephrectomy correlated with serum testosterone [26]. Glomerular enlargement is known to be linked with the occurrence of further injury of the aging kidney, e.g., glomerulosclerosis [27, 28]. Therefore, we conclude that in our animal model of neonatal uninephrectomy glomerular hypertrophy plays a crucial role in the sex specific differences of long-term renal damage. Accordingly, in our study UNX males showed more severe glomerulosclerosis compared to UNX females and controls. While Neugarten et al. did not find sex related differences of glomerulosclerosis in the human ageing kidney [29], in a rat model of uninephrectomy at 6 weeks of age simultaneous castration avoided the development of glomerular hypertrophy and secondary glomerular changes of aging specifically observed after uninephrectomy [30]. Further clinical and animal studies underline the deleterious role of male sex and the protective character of estrogen in diabetic kidney disease [31, 32].

In the KIMONO study, Westland et al. assessed the renal outcome of patients with congenital and acquired solitary functioning kidney (SFK). In both groups they reported an overweight of male patients with SKF. Consequently, the portion of male SKF patients suffering from renal sequelae was higher. However, statistical analysis did not prove a protective role of female sex in the development of secondary kidney injury [33]. Observing patients with congenital anomalies of the kidney and urinary tract (CAKUT), Wuhl et al. showed an earlier onset of end-stage renal disease in the male cohort [34].

We are not aware of previous studies of sex differences of kidney fibrosis following uninephrectomy under active nephrogenesis. Our data are in line with the notion of adverse effects of male sex in the development of glomerular alterations associated with aging. Considering the less severe renal injuries seen in females uninephrectomized during the vulnerable phase of organogenesis our study underlines the renoprotective role of female sex.

*Wt-1* and *nephrin* play crucial roles in podocyte development and maturation as well as in the maintenance of glomerular integrity [35, 36]. The expression levels of *wt-1* and *nephrin* were significantly reduced in both UNX females and males. One might assume that neonatal uninephrectomy during ongoing nephrogenesis disrupts signalling of those regulators of growth and differentiation. Moreover, expression levels of *wt-1* and *nephrin* of UNX males were found decreased compared to UNX females. Chau et al. showed that *Wt-1* and *nephrin* deficiency was associated with the development of glomerulosclerosis [37]. Against this background we speculate that the more severe glomerulosclerosis seen in UNX males might partly be caused by the more severe lack of *wt-1* and *nephrin* in these animals. Podocyte damage is accompanied with reduced expression of *wt-1* [38]. Accordingly, in UNX male rats our desmin score indicated more severe podocyte damage. Moreover, increased deposition of collagen I detected in renal tissue of UNX male rats argues for an induction of fibrosis in the remaining kidney.

Animal studies revealed the protective role of *Gdnf* in podocyte injury [39]. *Gdnf*-modified adipose-derived mesenchymal stem cells were found to attenuate glomerular fibrosis [40]. Itga8 is a well-known factor supporting glomerular homeostasis [41]. Expression levels of these genes were found increased in UNX females and may contribute to less severe renal fibrosis in those animals.

## Conclusions

Taken together, our data indicate a more severe kidney fibrosis in male animals 1 year after neonatal nephron loss under active nephrogenesis, compared to age-matched females. The detected alterations comprise signs of altered glomerular structure, podocyte damage, and renal interstitial fibrosis. We conclude that in preterm infants suffering from acute neonatal nephron loss male sex has to be regarded as an independent risk factor to develop secondary kidney disease later in life.

### Supplementary Information

**Additional file 1:** **Supplementary Table 1. **List of primers pairs and probes.

**Additional file 2:** **Supplemental Figure S1.**Glomerulosclerosis 52 weeks after neonatal uninephrectomy. Representative photomicrographs of PAS-stained glomeruli. UNX, rat uninephrectomized at day 1 of life. Sham, age-matched sham-operated control.

**Additional file 3: Supplemental Figure S2.** Interstitial fibrosis 52 weeks after neonatal uninephrectomy. Representative photomicrographs of Collagen I staining. UNX, rat uninephrectomized at day 1 of life. Sham, age-matched sham-operated control.

## Data Availability

All datasets generated and/or analyzed during the current study are available from the corresponding author on reasonable request.

## Abbreviations

ANOVA: Analysis of variance
Co: Age-matched sham-operated control
Gdnf: Glial cell line-derived neutrophic factor
Itga8: Integrin alpha-8
Mcp-1: Monocyte chemoattractant protein-1
MPF: Medium-power field
Kim-1: Kidney injury molecule-1
Ngal: Neutrophil gelatinase-associated lipocalin
PAS: Periodic acid-Schiff’s
Opn: Osteopontin
SEM: Standard error of the mean
UNX: Rat uninephrectomized at day 1 of life
Wt-1: Wilms’ tumor suppressor gene
