# Supplementary material for: Sex differences in long-term kidney fibrosis following neonatal nephron loss during ongoing nephrogenesis
Source: Mol Cell Pediatr. 2023 Aug 25;10:8. doi: 10.1186/s40348-023-00164-4 (PMC10457250; doi:10.1186/s40348-023-00164-4)
Supplement: Supplementary file 1 — Additional file 1: Supplementary Table 1. List of primers pairs and probes. [file 40348_2023_164_MOESM1_ESM.docx]

**Supplementary Table 1: List of primers pairs and probes**

|  | | **forward** | **reverse** |
| --- | --- | --- | --- |
| **18S** | 5’- TTG ATT AAG TCC CTG CCC TTT GT -3’ | | 5’- CGA TCC GAG GGC CTC ACT A -3’ |
| ***Ccl-2*** | 5’- CCTCCACCACTATGCAGGTCTC -3’ | | 5’- GCACGTGGATGCTACAGGC -3’ |
|  | 5’- TCACGCTTCTGGGCCTGTTGTTCA -3’ [Probe] | |  |
| ***Coll I*** | 5’- AGAGCGGAGAGTACTGGATCGA -3’ | | 5’- CTGACCTGTCTCCATGTTGCA -3’ |
| ***Coll IV*** | 5’- AACGAAAGGGACACGAGGA -3’ | | 5’- GGCCAGGAATACCAGGAAGT -3’ |
| ***Gdnf*** | 5‘-GGAACATGGAAGAAGAGTGTGAGA-3‘ | | 5‘-TCTGTGCCATTGCCAAAGG-3‘ |
| ***Itga8*** | 5‘-TCCAAATCAGAAGCTCCAACAA-3’ | | 5’-CGCTCACGAAATTGCTGTCA-3’ |
| ***Kim-1*** | 5’- ATAATCACACTGTAAGAATCCCTTTGAG -3’ | | 5’- CAACGGACATGCCAACATAGA -3’ |
| ***Nephrin*** | 5’- GGGGACCCCTCTATGATGAA -3’ | | 5’- GTGAAGCGTCTCACACCAGA -3’ |
| ***Ngal*** | 5’- TCACCCTGTACGGAAGAACCA -3’ | | 5’- ACTTGGCAAAGCTGACGAATC -3’ |
| ***Opn*** | 5’- AAAGTGGCTGAGTTTGGCAG -3’ | | 5’- AAGTGGCTACAGCATCTGAGTGT -3’ |
|  | 5’- TCAGAGGAGAAGGCGCATTACAGCA -3’ [Probe] | | |
| ***Synaptopodin*** | 5’- CGTAGCCAGGTGAGCCAAG -3’ | | 5’- GGCCCAAAGCTCCAGCA -3’ |
| ***Wt-1*** | 5’- AGGACTGCGAGAGAAGGTTTTCT -3’ | | 5’- TGGAATGGTTTCACACCTGTGT -3’ |
|  |  | |  |
|  |  | |  |
