# Supplementary figures and images for: Sex differences in long-term kidney fibrosis following neonatal nephron loss during ongoing nephrogenesis
Source: Mol Cell Pediatr. 2023 Aug 25;10:8. doi: 10.1186/s40348-023-00164-4 (PMC10457250; doi:10.1186/s40348-023-00164-4)

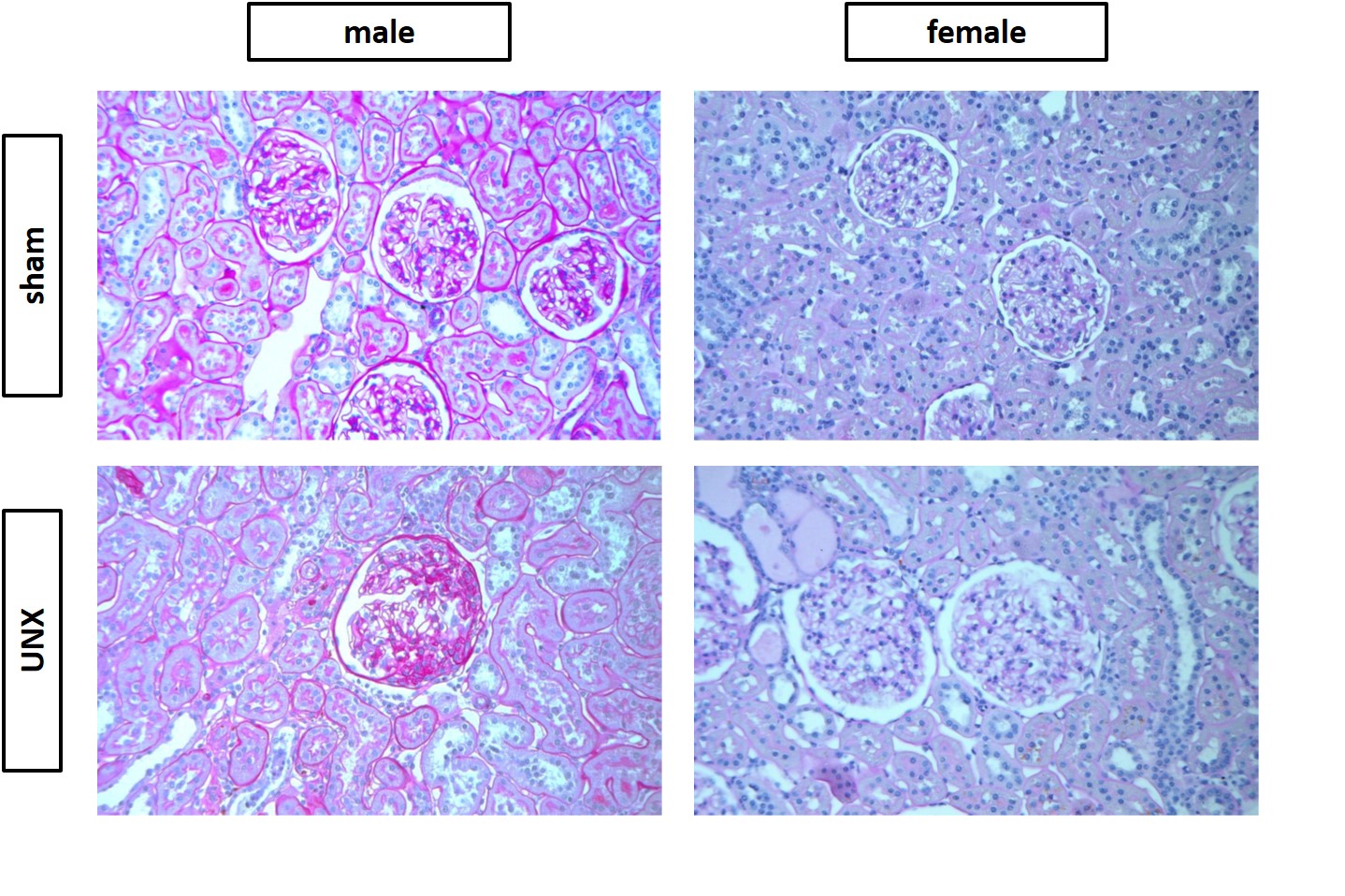

Supplement: Supplementary file 2 — Additional file 2: Supplemental Figure S1.Glomerulosclerosis 52 weeks after neonatal uninephrectomy. Representative photomicrographs of PAS-stained glomeruli. UNX, rat uninephrectomized at day 1 of life. Sham, age-matched sham-operated control. [file 40348_2023_164_MOESM2_ESM.jpg]

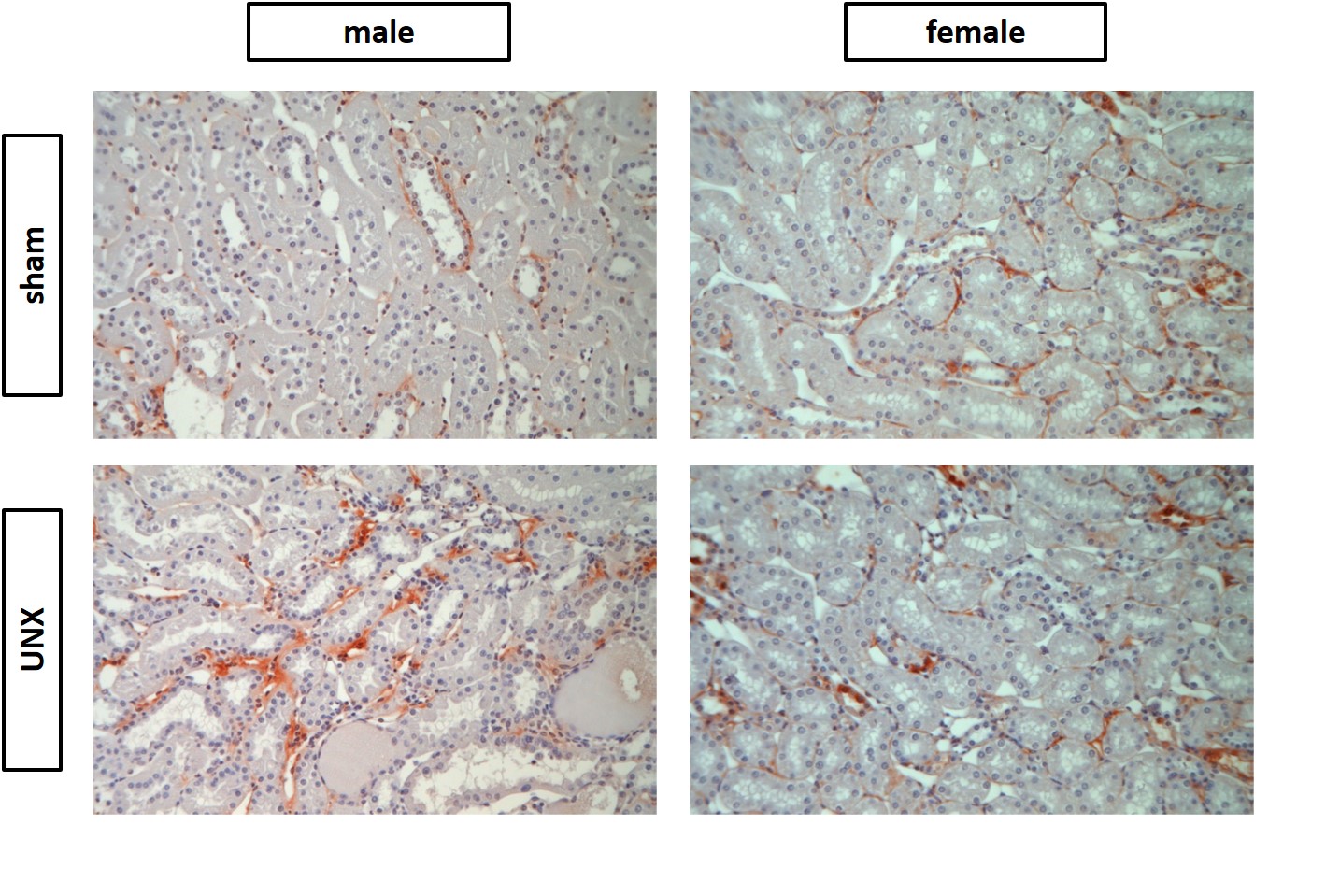

Supplement: Supplementary file 3 — Additional file 3: Supplemental Figure S2. Interstitial fibrosis 52 weeks after neonatal uninephrectomy. Representative photomicrographs of Collagen I staining. UNX, rat uninephrectomized at day 1 of life. Sham, age-matched sham-operated control. [file 40348_2023_164_MOESM3_ESM.jpg]
